# Pathogenic Molecular Mechanisms in Periodontitis and Peri-Implantitis: Role of Advanced Glycation End Products

**DOI:** 10.3390/life12020218

**Published:** 2022-01-30

**Authors:** Grigorios Plemmenos, Christina Piperi

**Affiliations:** 1 School of Dentistry, National and Kapodistrian University of Athens, Goudi, 11527 Athens, Greece; plemmenosgreg@windowslive.com or; 2 Department of Biological Chemistry, Medical School, National and Kapodistrian University of Athens, Goudi, 11527 Athens, Greece

**Keywords:** AGEs, periodontitis, RAGE, peri-implantitis, inflammation, PDL cells, therapy, diabetes

## Abstract

Advanced Glycation End Products (AGEs), the products of the non-enzymatic oxidation of proteins, nucleic acids, and lipids, are accumulated in periodontal tissues under hyperglycemic conditions such as Diabetes Mellitus (DM) and are responsible for sustained periodontal destruction. AGEs mediate their intracellular effects either directly or indirectly through receptor binding (via RAGE) in all types of periodontal ligament cells (osteocytes, gingival fibroblasts, stem cells, epithelial cells), indicating an important target for intervention. In combination with lipopolysaccharides (LPS) from *Porphyromonas gingivalis* (Pg), the negative impact of AGEs on periodontal tissue is further enhanced and accentuated. In addition, AGE accumulation is evident in peri-implantitis, yet through different underlying molecular mechanisms. Novel therapeutic approaches targeting the effects of AGEs in periodontal ligament cells show beneficial effects in pre-clinical studies. Herein, we provide evidence on the detrimental role of AGE accumulation in oral cavity tissues and their associated signaling pathways in periodontitis and peri-implantitis to further highlight the significance of oral or topical use of AGE blockers or inhibitors along with dental biofilms’ removal and DM regulation in patients’ management.

## 1. Introduction

**Figure 1:**
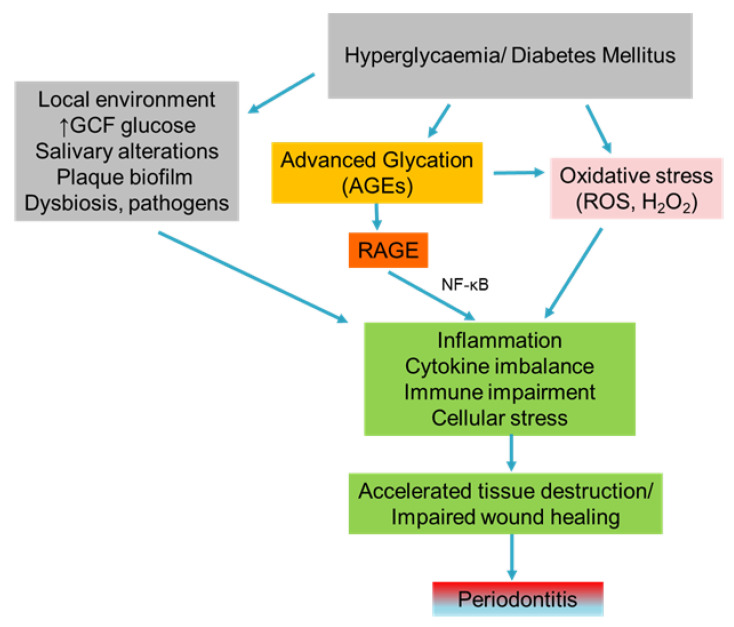
Sequences of events that lead to periodontitis in hyperglycemic or diabetic conditions mediated by increased levels of AGEs and oxidative stress in combination with periodontal pathogens.

Periodontal disease is mainly attributed to poor oral hygiene along with a plethora of inherited and environmental factors. A deep understanding of these factors and the associated molecular mechanisms is required to prevent or attenuate periodontal lesions [1,2]. The initial phase of periodontal inflammation is gingivitis [3], and the transition to periodontitis relies on several factors, including the shift of aerobic Gram-positive bacterial species dental plaque to anaerobic Gram-negative, genetic alterations, and host environment factors. Although microbial agents present the etiological factors of periodontal disease, with a direct effect in oral tissues, they also mediate harmful inflammatory reactions in the susceptible host through the formation of strongly adhesive biofilms at the tooth surfaces [4]. Metabolic disorders characterized by hyperglycemia and insulin resistance, mainly Diabetes Mellitus (DM), are responsible for alterations of oral microbiota. The reduced diversity of bacteria has been observed in diabetic mice, with Proteobacteria and Firmicutes being the prevailing species. This effect was only partially reversed with the administration of antibodies against the pro-inflammatory cytokine IL-17 as well as against the inflammatory mediators RANKL and IL-6, indicating a strong correlation between inflammation, oral bacterial composition, and DM (Figure 1) [5]. 

The magnitude of host periodontal cells’ immune response on confronting the activity of pathogens is very important because it regulates tissue resistance and host susceptibility and determines the clinical outcome of periodontitis. Some critical inflammatory mediators such as IL-1β, TNF-α, prostaglandin E2, and metalloproteinases (MMPs) play a significant role in the immune response, which is mainly regulated by the T helper cells. Periodontal pathogens have been shown to activate the innate immune system through the production of inflammatory mediators and contribute to the progression of periodontal disease [6]. 

Upon extended inflammation, the attachment of the junctional epithelium to the root surface is disrupted, forming a periodontal pocket, which is colonized by multiplying bacteria. In this new environment, bacteria release toxins as the byproducts of their metabolism and further contribute to alveolar bone loss [7]. In established periodontal disease, the underlying mechanisms of the host-mediated response involve the activation of innate immunity through the upregulation of pro-inflammatory cytokines in the presence of sub- and upper-gingival bacterial biofilms [4]. Clinically, bleeding gingiva and attachment loss due to the formation of periodontal pockets represent the first signs of periodontitis [6]. Lately, a new classification of periodontal diseases has been proposed, which includes a two-vector system. Upon the initial diagnosis of periodontitis, the stage and the grade of the periodontal disease need to be determined. The stage refers to the severity of periodontal destruction and is further divided into four stages, with stage I referring to the initiation of periodontitis and stage IV to the advanced periodontitis with minimal possibilities of dentition preservation. On the other hand, grade allocation (grades A, B, C) refers to the possibility of an anticipated response of the patient to traditional periodontal treatment strategies. Risk factors that worsen the grade and that influence the therapy of periodontitis include smoking, diabetes, and chronic inflammatory diseases [8].

Over the years, periodontitis has been associated with several systemic conditions, including DM, rheumatoid arthritis, atherosclerosis, metabolic syndrome, inflammatory bowel disease, chronic kidney disease, respiratory disease, Alzheimer’s disease, and low birth weight [6]. Among these systemic diseases, DM has been directly correlated to periodontal disease as it can enhance inflammation by elevating the expression of pro-inflammatory cytokines (IL-1β, IL-6, IL-8, IL-17, TNF-α) and significantly reduce the levels of anti-inflammatory mediators (TGF-β, IL-4, IL-10). The chronic persistence of high glucose levels induces cytokines’ overexpression and, consequently, the formation of reactive oxygen species (ROS) with or without the involvement of enzymes (Figure 1) [5]. Lately, the research field of DM epigenetic alterations on periodontitis has gained great attention. An in vivo study using a pig model of DM has demonstrated that hyperglycemic status is responsible for the epigenetic modification of 1163 genes in gingival tissues. These epigenetic modifications were further accompanied by histological alterations, including acanthosis of squamous and junctional epithelium. Additionally, the hypomethylation of *TNF-α* and *IL-6* genes was shown to induce their overexpression at the junctional epithelium [9].

Current data suggest that the irreversible accumulation of AGEs in periodontal tissues because of DM leads to the deterioration of periodontal status in diabetic subjects (Figure 1) [10]. A correlation between DM duration and AGE levels has been proposed, with long-term diabetic states being positively correlated with higher AGE levels in gingival tissues (epithelium, vessels, and fibroblasts). Consequently, Type 1 Diabetes Mellitus (T1DM) patients exhibited greater AGE levels compared to Type 2 (T2DM) patients and controls. In the same clinical trial, HbA1c was not regarded as a marker of AGEs’ presence in gingiva because it only depicts recent glucose concentrations (of some months ago) and not the long-lasting exposure to metabolic aberrations of DM patients [11]. Currently, there is no adequate evidence in respect of the reverse role of periodontal status and periodontal treatment on DM control at a biological level. The lack of a great number of animal studies focusing on the mechanisms through which periodontitis may deregulate glucose homeostasis has already been stated [12]. Nonetheless, an animal study showed that the presence of the lipopolysaccharide of *Porphyromonas gingivalis* (Pg LPS) in gingival tissues impaired the glucose metabolism in mice receiving a high-fat diet by modulating the regional adaptive immune system. A high-fat diet further reduced anti-Pg antibodies and subsequently led to insulin resistance. Therefore, Pg abundance in oral microbiota was positively correlated with glucose intolerance as the continuous infusion of Pg LPS for one month along with a high-fat diet impaired the glucose intolerance in mice [13].

Contradictory results exist in relation to the potential positive effect of periodontal treatment on DM control, further questioning the therapeutic approach of reversing high glucose levels by using periodontal intervention [12]. 

**Figure 2:**
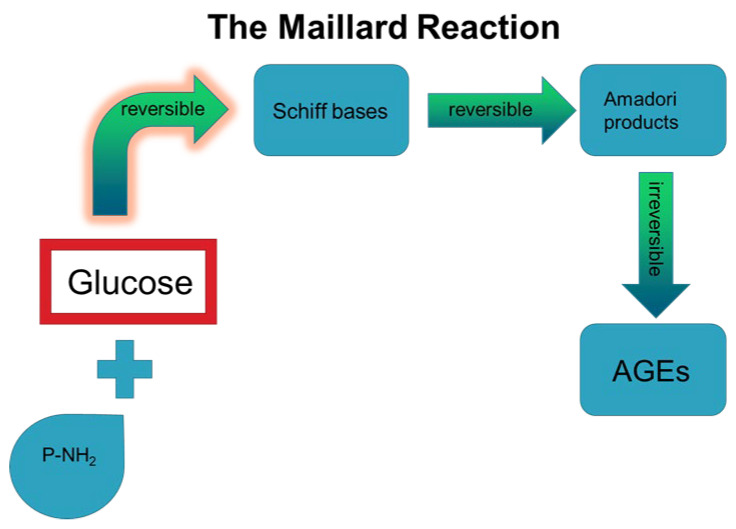
The steps of Maillard reaction that result in irreversible AGE formation.

Glycation reaction, also known as Maillard reaction, refers to the formation of a covalent bond between the carbonyl group of sugars, such as glucose and fructose, and the free amino group of proteins or lipids or nucleic acids, without enzymatic involvement (Figure 2) [14]. After a short time, the reversible Schiff bases that are formed by glycation induce a series of chemical rearrangements, producing Amadori products [15]. Although a reduction in glucose levels may reverse the formation of Amadori products, their persistent formation and presence will enhance rearrangement reactions, including dehydration and redox, resulting in stable compounds, known as Advanced Glycation End Products (AGEs) (Figure 2) [16]. Alternatively, auto-oxidation of Amadori products may induce the production of toxic AGE compounds [15]. Upon formation, AGEs can be distributed through the circulation to all the peripheral tissues where they localize, exerting both direct and indirect effects. In the following sections, we describe the experimental evidence on the direct and indirect effects of AGEs in the main cell types of the periodontal ligament in an effort to highlight their importance in disease management. It is plausible that the administration of AGE inhibitors may improve the outcome of the periodontal treatment and therefore contribute to the reduction in inflammation and enhanced wound healing of periodontal tissues. The great potential of this approach is evident by the application of novel agents such as vitamin C [17] and magnolol [18] in periodontal tissues, exerting a beneficial action by diminishing AGE-induced oxidative stress. 

In the following sections, we provide recent experimental and clinical evidence on the direct and indirect effects of AGEs in the different types of periodontal ligament cells in an effort to exploit the underlying molecular mechanisms that contribute to their pathogenic function and point out targeted therapeutic approaches.

The literature search was performed within PubMed and Scopus databases using the following keywords in various combinations: ‘advanced glycation end products’, ‘AGEs’, ‘DM’, ‘RAGE’, ‘periodontitis’, ‘peri-implantitis’, ‘PDL’, and ‘inflammation’. Both original pre-clinical and clinical studies as well as reviews, published in the English language, to December 2021 were included.

## 2. Direct Effects of AGEs in Periodontal Ligament Cells

### 2.1. AGE Effects on Osteoblasts-Osteocytes

#### 2.1.1. In Vitro and In Vivo Studies

AGEs have been demonstrated to affect bone metabolism by directly cross-linking to structural proteins, decreasing the expression of type 1 collagen, the core-binding factor alpha 1, and osteocalcin, while increasing IL-1β and calcium-binding protein, S1008A. These actions were shown to be aggravated by the co-injection of Pg LPS, which can also downregulate osteocalcin levels. Additionally, AGEs’ presence in combination with Pg attenuated alkaline phosphatase (ALP) activity in rat bone marrow cells, a known biomarker of osteoblastic activity [19]. Another potential action of AGEs is the 19-fold upregulation of sclerostin, which has a negative impact on bone formation in osteocyte-like cells and is further accentuated by the presence of Pg LPS (up to 30-fold). Increased expression of Toll-like receptor 2 (TLR2) in osteocytes was observed after Pg incubation, which was further enhanced by the synergistic action of AGE accumulation and Pg LPS. The functions of AGEs were shown to be mediated by RAGE, ERK/JNK, and NF-κB signaling pathways in the osteocytes, while AGEs’ presence upregulated IL-6 and TNF-α levels [20]. The same study demonstrated a positive correlation of osteoblastic cells co-cultured with the osteocytes with ALP activity and Runx2 expression [20]. More specifically, sclerostin was shown to downregulate Runx2 expression and ALP activity as the inhibition of sclerotin with a specific neutralizing antibody sufficiently reversed the sclerostin-mediated bone resorptive effects [20].

Exogenous sources of AGEs may enhance the endogenous metabolic processes and substantially increase the circulatory AGE levels. The most established sources of exogenous AGEs are high-glucose content and heat-processed foods, soft-drinks, flavored beverages, and tobacco smoking, all contributing to the detrimental effects of accumulated AGEs in periodontal tissues [21]. An in vivo study of rats fed on a high-fat diet or normal chow showed a higher accumulation of AGEs in the serum of the high-fat diet-fed rats, which was associated with increased inflammation in the periodontal tissue cells, further suggesting an association between a non-balanced diet and AGE accumulation [22]. 

Another exogenous factor that contributes to endogenous metabolic processes is nicotine. In favor of this hypothesis, a study showed that nicotine administration in human osteoblasts induced cell apoptosis [23]. In more detail, nicotine was shown to induce the intracellular accumulation of H_2_O_2_, which blocks glyoxalase 1 (Glo1), a detoxification enzyme of the main AGE precursor, methylglyoxal (MG). It further amplified methylglyoxal-derived hydroimidazolone (MG-H1), a key AGE compound derived from the release of arginine residues. Following MG-H1 release, H_2_O_2_ participates in a mechanism of a positive feedback loop by MG-H1 via RAGE, thus inducing its overexpression. Moreover, the NF-κB pathway was shown to be desensitized independently of RAGE inhibition but rather through transglutaminase 2, involving an apoptotic mitochondrial pathway [23]. 

#### 2.1.2. Clinical Studies

Periodontal patients with T2DM exhibit a significantly higher concentration of AGEs in gingival crevicular fluid (GCF) compared to the normoglycemic non-periodontal group, indicating a severe negative impact of DM to PDL health [24]. A clinical trial that evaluated the biomarker potential of AGEs in GCF failed to detect a statistically significant difference between T1DM patients and healthy subjects. However, the main limitation of this study was the exclusion of patients with uncontrolled DM as well as those with severe periodontitis [25]. 

Patients undergoing fixed orthodontic treatment with T2DM exhibited significantly higher levels of resistin and AGEs in their GCF than those without T2DM. The rest of the inflammatory markers (TNF-α, IL-6, RANKL, and Ghrelin) under investigation were increased but this was without reaching statistical significance between the two groups [26]. 

### 2.2. AGE Effects in Human Gingival Fibroblasts (HGFs)

#### 2.2.1. In Vitro Studies

Experimental evidence indicates that the transcription of collagen type I and III was suppressed in the presence of AGEs in HGF cells [27,28]. The inhibitory influence of AGEs on the expression of type I collagen mRNA as well as on HGF proliferation was demonstrated in periodontium [28]. Interestingly, the glycation of fibronectin and type I collagen led to the modification of HGF and PDL fibroblast responses. Fibronectin glycation resulted in reduced cell attachment while fibronectin and type I collagen glycation significantly altered cell migration [29].

Moreover, studies showed that HGF cell viability was reduced after the incubation of AGEs in a dose-dependent manner. The cell death rate was increased after the administration of D-glucose, indicating the direct effect of AGEs on HGFs and the deleterious role of hyperglycemia in cell proliferation [27]. The apoptosis of HGF was increased by AGE-human serum albumin (HSA), possibly mediated by elevated oxidative stress [30].

High glucose levels in combination with the exogenous administration of AGEs induce upregulation of pro-inflammatory cytokines IL-6 and IL-8 in HGFs. Further secretion of IL-8 was detected in the presence of Pg LPS, highlighting an underlying synergistic mechanism [21]. Glycated-human serum albumin (G-HSA), a representative form of AGEs, was not cytotoxic for HGFs. IL-6, IL-8, and monocyte chemoattractant protein-1 (MCP-1) levels were significantly increased in the group treated with G-HAS, and the combination of the Pg LPS further increased the cytokine amounts. Likewise, the gene expression of MMP-1, MCP-1, IL-6, and IL-8 was augmented while the IL-1β and TNF-α levels did not show any significant alterations. Moreover, the synergistic action of Pg LPS and G-HSA in the activation of the NF-κΒ pathway was confirmed [31].

Similar findings were observed upon the addition of high glucose concentrations in HGFs, which induced AGE formation and upregulated the expression of IL-6, RAGE, and intracellular adhesion molecule-1 (ICAM-1) in these cells [32]. The combination of AGEs with Pg LPS was shown to exacerbate inflammatory responses (through elevation of IL-6 and ROS production). The expression of ICAM-1 was elevated in an IL-6-dependent manner, while RAGE, MAPK, and NF-κB pathway activation was AGE-dependent [32].

#### 2.2.2. In Vivo Studies

A study investigating the role of AGEs in rhesus monkeys revealed that the gradual increase in AGE levels in serum paralleled with the progression of DM and was positively correlated with fasting blood glucose levels [33]. The underlying mechanisms involve the attenuation of the growth of HGFs and the secretion of MMP-1. Downstream signaling of AGEs has been shown to increase the expression of IL-17 mRNA in gingiva and downregulate the mRNA levels of a salt insensitive host defense peptide, beta-defensine-3 [33]. In addition, AGEs’ presence enhanced the cellular oxidative stress by activating the NF-κB transcription factor and increasing the IL-6 mRNA levels, which is a pro-inflammatory mediator in periodontitis [34]. Furthermore, AGEs efficiently increased CSF-1 and glucose transporter-1 (GLUT-1) expression in PDL fibroblastic cells while VEGF levels remained stable. When tension was applied, only CSF-1 levels were significantly increased in AGE-treated rats compared to normoglycemic animals. On the other hand, compression forces were shown to increase the levels of VEGF, CSF-1, and GLUT-1 expression. This upregulation was also maintained in rats incubated with AGEs and was mediated by JNK and p38 signaling pathways. AGE receptor 1 (AGE-R1) silencing led to the reduction in CSF-1 and VEGF overexpression, even though the latter was not induced by AGEs as it was mainly expressed in PDL vascular cells rather than in fibroblastic cells [35]. 

### 2.3. AGE Effects in hPDL Cells

#### In Vitro Studies

**Figure 3:**
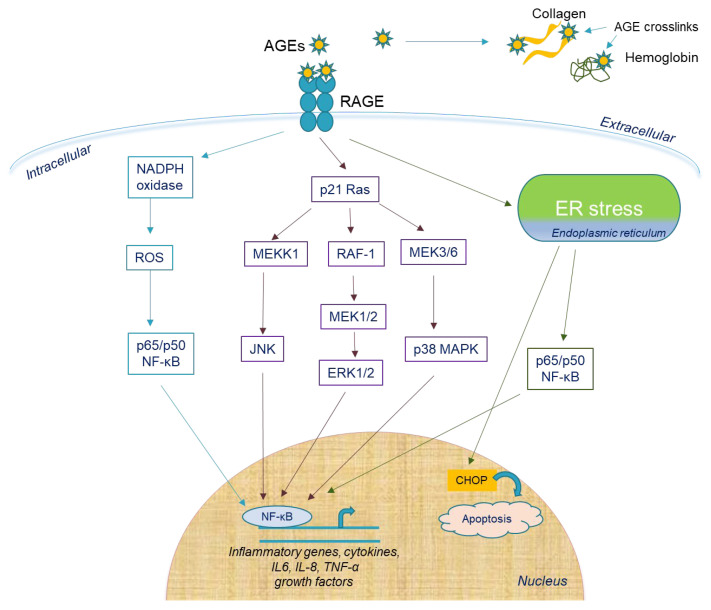
Extracellular and intracellular effects of AGEs in periodontal tissue. AGEs affect bone metabolism through direct cross-linking to structural proteins, such as type 1 collagen, and osteocalcin. AGE accumulation leads to protein misfolding and induces endoplasmic reticulum (ER) stress in hPDL cells, which may further activate NF-κB to increase inflammatory gene transcription or upregulate CHOP expression to induce apoptosis. AGE–RAGE binding increases the activity of NADPH oxidase to induce ROS production and NF-kB activation. Alternatively, p21 Ras activation induces NF-κB activation through JNK, ERK1/2, or p38MAPK upregulation, leading to increased transcription of inflammatory mediators, cytokines, growth factors, etc.

A low concentration of AGEs (<100 μg/mL) did not induce cellular damage after a short time, while a higher concentration (>150 μg/mL) exhibited a direct negative effect on hPDL cells. Inflammation induction through endoplasmic reticulum stress (ERS) was mediated by AGEs in concentrations greater than 50 mg/mL, suggesting their potent inflammatory role. ERS was induced by the upregulation of ERS chaperones (GRP78, PERK, ATF-6), unfolded protein response (UPR) stimulation through caspase 12 and CCAAT/enhancer-binding protein homologous protein (CHOP) and increased nuclear translocation of NF-κB-p65, leading to IL-6 and IL-8 production (Figure 3) [36]. In vitro data showed that in hPDL cells, the receptor that mediated S100A9 function was TLR4. The S100A9-induced release of IL-6 and IL-8 was mediated by the NF-κB and p38 MAPK signaling pathways, while IL-8 release involved the ERK1/2 and JNK 1/2 pathways [31]. Lastly, ROS exerted a large regulatory role on the S100A9-induced secretion of IL-6 and IL-8 [37]. Moreover, ROS production during AGE-induced apoptosis of hPDL cells through the depolarization of the mitochondrial membrane was shown to participate in the autophagy of hPDL cells. Furthermore, the microtubule-associated protein 1A/1B-light chain 3II (LC3-II) conversion was increased and p62 was downregulated via the ERK pathway, indicating that ROS enhances autophagy via the activation of the ERK pathway at the presence of AGEs. In this way, ROS-induced hPDL cells’ autophagy could be a new therapeutic target for DM patients with periodontitis [38].

### 2.4. AGE Effects in Gingival Epithelial Cells

#### In Vitro Studies

Studies using the human gingival epithelial cell line OBA-9 demonstrated that the AGEs and Pg LPS combination upregulated the production of calprotectin and its heterodimers, S100A8 and S100A9, through JNK, p38, MAPK, and NF-κB pathways. Calprotectin was detected at high concentrations in the neutrophils’ cytosol. However, the fact that S100A8 and S100A9 were increased may lead to the conclusion that these proteins may also act separately. In accordance, S1008A immunoreactivity in the nucleus was increased while S100A9 remained unchanged. Of note, AGEs and Pg LPS demonstrated synergistic effects, enhancing the formation of these proteins. However, there is also contradictory evidence indicating that AGEs increased the expression of IL-6 mRNA without exerting any effects in IL-8 mRNA levels in gingival epithelial cells, as previously observed [37,39]. AGEs were shown to upregulate the expression of its main ligand RAGE in OBA-9 cells and this action was co-amplified by Pg LPS. The blockage of RAGE with specific siRNA only partially inhibited the expression of S100A8 and S100A9, indicating that AGEs may also exert direct effects in OBA-9 cells [39]. 

### 2.5. AGEs Effects in hPDL Stem Cells (hPDLSCs)

#### In Vitro Studies

AGEs were shown to significantly induce the formation of oxidative stress and increase endogenous ROS production in hPDLSCs in vitro. The long-term accumulation of AGEs resulted in the maintenance of high ROS levels, leading to the activation of the JNK pathway and the consequent mitochondria-mediated apoptotic signals. These signals include increased Bax expression, intracellular Ca^2+^ concentrations due to increased membrane permeability, caspase-3, and decreased B-cell lymphoma 2 (Bcl-2) protein levels, along with mitochondrial membrane depolarization, an early stage of apoptosis. TNF-α in synergy with AGEs exhibited a greater damaging effect on hPDLSCs [40]. The osteogenic nodules formed in hPDLSCs of the AGE-stimulated group were significantly less calcified and the expression of the corresponding genes was notably lower. This observation can be attributed to the stimulation of the Wnt signaling pathway as the inhibitor dickkopf-1 (DKK-1) was significantly lower in the AGE-stimulated cell group [41]. AGEs were suggested to block osteogenic differentiation of hPDLSCs by modulating the expression of microRNA-17. Regarding the underlying mechanisms, bone sialoprotein, ALP, and Runx2 expression levels in the AGE-stimulated experimental group were found to be remarkably downregulated [42]. Further studies showed that AGEs demonstrate a partially inhibitory effect to the differentiation of hPDLSCs, resulting in lower osteogenic potential and alveolar bone repair, possibly through the activation of the canonical Wnt/β-catenin pathway. The use of berberine hydrochloride, a traditional Chinese drug, was shown to reverse the reduction in the osteogenic differentiation potential of hPDLSCs in vitro, partially through the Wnt/β-catenin signaling axis. Further investigation of Berberine’s additional targets would enable a better understanding of the role of AGEs in diabetic-associated periodontitis [43].

### 2.6. AGE Effects in THP-1 Cells

*Tannerella Forsythia*, a bacterial species that usually colonizes the oral cavity, has been shown to produce methylgloxal, a precursor of AGEs that further activates the NF-κΒ pathway, eliciting the secretion of pro-inflammatory mediators, such as IL-1β and TNF-α in THP-1 cells in vitro [44]. 

## 3. Indirect Effects of AGEs in Periodontal Ligament Cells Mediated through Receptors

Although RAGE was at first believed to be an exclusive receptor for AGEs, subsequent research detected additional receptors, including scavenger receptor types I and II, oligosaccharyl transferase-48 (OST-48, AGE-R1), 80K-H phosphoprotein (AGE-R2), and galectin-3 (AGE-R3) [45]. Moreover, AGEs have been shown to bind to mammalian lactoferrin (Lf) receptor 1 (Lf1) as well as to RAGE in gingiva [46]. The administration of a soluble form of RAGE (sRAGE) was shown to act as an anti-inflammatory agent in a dose-dependent manner by preventing the activation of full-length RAGE and reducing alveolar bone resorption in a diabetic rat model. Notably, the enhancing effect of sRAGE was independent of glucose levels, suggesting that the attempt of reducing periodontal inflammation is not influenced by diabetes. However, because sRAGE was administered intraperitoneally, further research was required to focus on the local application of sRAGE on periodontal tissues that may lead to inflammation control [47]. Furthermore, the systemic administration of aminoguanidine (an anti-AGE agent) was effective in reducing inflammatory responses, more likely by blocking the AGE–RAGE axis, although the osteogenic process was not significantly enhanced. Micro-computed tomography parameters showed no remarkable differences 7 days after the administration of AG, and a histological assessment of the newly formed bone differed compared to the native bone at the fibril arrangement, probably due to local hypoglycemia [48].

Elevation of RAGE and AGE levels was observed in the non-diabetic periodontal group, indicating the upstream role of inflammation and oxidative stress to AGE production [49]. AGEs in high concentrations have been proposed to act as endogenous ‘danger signals’ and, upon binding to RAGE, to lead to the activation of the TLR/MyD88/NF-κB pathway, further exacerbating the inflammatory response. To further elucidate the underlying mechanisms, the TLR-2 and -4 expressions were upregulated by the AGE–RAGE axis, inducing MyD88 signaling. This further activates the inflammatory mediator NF-κB pathway, mediating the secretion of pro-inflammatory cytokines, including IL-1 and TNF-α. Of note, no significant alterations were observed on the dental biofilms of the diabetic rats [50]. Ιn a study of a south Indian population, it was shown that RAGEG82S gene polymorphism could be a risk factor for periodontal disease only when DM co-exists [51]. Another possible action of the AGE–RAGE axis is the reduction in dendritic cell migration, observed in vivo [52]. 

In diabetic patients, salivary RAGE detection has been suggested as a novel biomarker of periodontal health. Serum AGE levels were reported to be elevated in systemically healthy periodontal patients compared to the non-periodontal group, shedding light on the yet not fully known correlation between periodontitis and AGEs [53]. Serum and GCF concentrations of sRAGE were reduced in patients with periodontitis and T2DM compared to those without T2DM. On the same study, TNF-α was controversially elevated, suggesting an antagonistic role of these two molecules and their potential role as valuable biomarkers for high-risk patients [54]. sRAGE levels were found to be downregulated in serum, possibly due to enhanced binding to AGE ligands [53].

### 3.1. AGE–RAGE Axis in HGFs

#### In Vitro Studies

High doses of AGEs were shown to increase the levels of the inflammatory mediator MMP-1 in a dose-dependent manner and a long incubation time was detrimental for the viability of HGFs. Moreover, in periodontal connective tissues, RAGE and NF-κB mRNA levels were significantly increased in T2DM patients when compared to healthy controls [55]. This dependence of AGE effects on dose and time in HGFs confirms the hypothesis that T1DM patients, exposed to hyperglycemia for additional years, present a more deteriorating clinical outcome. In accordance, an in vitro study investigating the potential pathogenic mechanisms that occur in periodontitis associated with DM showed that exposure of human PDL fibroblastic cells to AGE-BSA induced upregulation of RAGE and cell apoptosis via the AGE–RAGE axis [56].

### 3.2. AGE–RAGE Axis in hPDL Cells

#### In Vitro Studies

Increased levels of RAGE and TLRs have been detected in cells seeded on a glycated matrix followed by the activation of the NF-κΒ pathway. Pg LPS possibly acts via RAGE in periodontitis, in parallel with the AGE–RAGE axis, sharing a common signaling pathway and further confirming the synergistic effect of Pg LPS with AGEs that has previously been proposed. Compared to mesenchymal stem cells, hPDL cells exhibited smaller sensitivity to Pg LPS and high glucose concentrations [57]. 

Another study detected increased IL-6, IL-1β, NLRP1, and NLRP3 after stimulation of hPDLs by AGEs, while nucleotide-binding oligomerization domain-containing protein 2 (NOD2) levels remained unaltered. The silencing of *RAGE* reversed the AGE-induced upregulation of NLRP1- and NLRP3- inflammasome. After treatment with a specific NF-κB inhibitor, the levels of IL-6 and IL-1β returned to similar concentrations with the control group. Moreover, co-stimulation of the hPDL cells with AGEs and muramyl dipeptide resulted in an upregulation of NLRP1, NLRP3, and NOD2, suggesting the potential involvement of those factors in the increased production of IL-1β and IL-6 [58].

### 3.3. AGE–RAGE Axis in Epithelial Cells

AGEs were shown to upregulate the levels of neutrophil gelatinase-associated lipocalin (LCN2) in a RAGE-dependent manner in vitro. The inhibition of RAGE with specific anti-RAGE antibodies sufficiently reversed this effect in oral epithelial cells. Among LCN2 actions was the indirect inhibition of IL-6 expression in neutrophils, while at the same time, it directly enhanced their migration. Upon incubation with AGEs, LCN2 modulated inflammatory responses via RAGE and by activating NF-κB and MAPK pathways, indicating a potential role in the pathogenesis of periodontitis associated with DM [59]. 

A clinical study reported the significantly increased mRNA levels of RAGE in the gingiva of DM patients with periodontitis [60]. Furthermore, RAGE was shown to mainly be localized on the basal epithelial membrane, as well as on the stratum spinosum, and was expressed in both diabetic and non-diabetic periodontal patients [61]. Hyperglycemia associated with T2DM was demonstrated to increase the availability of TLR and RAGE on the cell surface of oral epithelial cells. As TLR4 and RAGE lie upstream of the pro-inflammatory cytokine response, the administration of selective TLR4 and RAGE antagonists could have therapeutic potential in diabetic patients [4].

### 3.4. AGE–RAGE Axis in Gingival Tissues 

In clinical studies, AGER1 has been proposed to efficiently block AGEs, thus attenuating oxidative stress and inflammation. A clinical trial investigated the role of RAGE and AGER1 in gingival tissues and showed that in diabetic periodontal patients, AGER1 was less efficient in downregulating the activation of RAGE, probably due to its longstanding interaction with AGEs and oxidative stress. However, in non-diabetic periodontal patients, AGER1 was shown to impede RAGE activation only partially [62,63].

Additionally, gingival biopsies taken by smokers were found to contain RAGE in higher concentrations than the control healthy group, suggesting that smoking has a direct impact on RAGE expression. In accordance, an upregulation of RAGE expression on epithelial cells of the gingiva was observed after incubation with nornicotine, a nicotine metabolite [64]. Moreover, RAGE levels in gingival biopsies from clinically healthy edentulous ridges were found elevated in association with the higher protein expression in the periodontal resident cells rather than in infiltrating inflammatory cells [65]. By using RAGE-specific antibodies, an increased expression of RAGE was observed in the gingiva of DM patients compared to the healthy gingiva of the control group. Additionally, the non-diabetic periodontitis group exhibited lower levels of RAGE expression and of inflammatory cell numbers than the diabetic periodontitis patients [60]. The immunoreactivity for RAGE was also significantly higher in the T2DM group compared to the control without a systemic disease. In the same clinical trial, aging was suggested as a risk factor of periodontitis due to the linear increase in RAGE expression in gingival biopsies with patients’ chronological age [46]. However, this hypothesis was not confirmed in another study, which proposed that the disease duration, and not the age, was associated with increased risk for periodontitis [53]. Another conclusion of this study was the positive correlation between HbA1c levels and RAGE, which, however, is not consistent among studies [11,46]. 

### 3.5. AGE–RAGE Axis and Periradicular Tissues

In a small clinical study, it was shown that inflaming peri radicular tissues because of endodontic infection presented increased levels of RAGE mRNA expression and upregulated NF-κB mRNA levels [66]. 

### 3.6. AGE–RAGE Axis in Endothelial Cells

The correlation between the AGE–RAGE axis and periodontitis was further investigated in human and murine aortic endothelial cells in vitro. The results revealed amplified inflammatory responses in endothelial cells infected with Pg, and this effect was mediated by the AGE–RAGE axis as inhibition of AGEs and ROS by AG and antioxidant treatment (N-acetylcysteine, diphenylene iodonium) was shown to suppress MCP-1 production and prove beneficial for the cells [67]. The upregulation of vascular cell adhesion molecule-1 (VCAM-1) via AGE–RAGE signaling has been further detected in diabetic vasculopathy, a condition often observed in the gingival vessels of DM patients [68]. However, the underlying mechanisms in periodontal vessels have not been revealed yet. 

## 4. Effects of AGEs in Peri-Implantitis

The two major risk factors for periodontal and peri-implant diseases are DM and smoking. Although these two factors are well studied with periodontitis, in regard to peri-implantitis, the knowledge is more limited. Current research is focused on shedding light on the exact mechanisms and the different influence of smoking and DM on peri-implant tissues. It has been observed that hyperglycemia rather than smoking induces inflammation in peri-implantitis, and at the absence of both nicotine and high glucose, smaller inflammatory responses are elicited [69]. Metanalysis data showed a 50% greater risk for peri-implantitis between diabetic and normoglycemic patients, while non-smoking diabetics exhibited almost 3.5-fold greater risk in comparison with non-smoking non-diabetic controls, indicating that hyperglycemia is an independent high-risk factor for peri-implantitis. Surprisingly, no correlation was observed in terms of peri-implant mucositis among all patient groups [70].

Based on the accumulating evidence that AGEs and ROS are important mediators of gingival and periodontal inflammation, a crucial question arises about their involvement in peri-implant diseases. Periodontal patients presented significantly higher values of oxidative stress (measured through lipid peroxidation) and AGEs compared to patients suffering from peri-implantitis, while both had higher values than healthy individuals [71]. In accordance with these results, a strong correlation between peri-implantitis and oxidative stress conditions related to AGEs was confirmed in a study of peri-implantitis, periodontitis patients, and healthy subjects [72]. AGE levels were statistically increased in the peri-implantitis group compared to healthy controls, while the highest levels among all three were observed in the periodontitis group [72]. Another approach to investigating the role of AGEs in peri-implantitis included the evaluation of AGE levels and clinical parameters in T2DM patients with different concentrations of HbA1c. In the group with HbA1c values greater than 10%, bleeding on probing and probing depth were positively correlated with AGE levels. Additional findings indicate the statistically higher AGE levels in the diabetic patients with HbA1c greater than 10% and the control group as well as the group with HbA1c values between 8.1% and 10% [73]. These data suggest that the pathogenic mechanisms may differ between periodontitis and peri-implantitis and may be attributed to the absence of periodontal ligament in peri-implantitis. 

Moreover, an investigation of RAGE and TLR4 protein expression in peri-implant tissues of periodontal-susceptible participants showed a significant upregulation of their expression two months after the implant installation, while at the same sites TLR2 expression was significantly reduced. The opposite findings were observed in periodontally healthy subjects with implants, with TLR2 levels being elevated while TLR4 and RAGE being reduced [65]. The reasons for these contradictory results remain unclear. Furthermore, AGE levels are positively correlated with probing depth and marginal bone loss in T2DM patients but only with probing depth in prediabetic patients, while the highest concentration of AGEs was detected in the peri-implant sulcular fluid (PISF) of T2DM patients compared to healthy and prediabetic ones. It is noteworthy that the difference in AGE levels between the prediabetic and diabetic patients could be attributed to the shorter mean duration of prediabetes in those patients, which was approximately 3 years less than the average 8-year disease duration of the T2DM patients [74]. Considering that AGE–RAGE signaling enhances inflammation in periodontitis [32,54], a clinical trial was carried out to investigate the role of AGEs in peri-implant diseases, which are sub-grouped based on the extension of inflammation to peri-implant mucositis and peri-implantitis. Predictably, AGEs in PISF were detected to be fourfold higher in smokers compared to non-smokers, suggesting that nicotine is responsible for the upregulation of AGEs [75]. These data explain the worst clinical outcome of non-smokers in plaque, gingival index, and probing depth, suggesting that AGEs present a threat to the osseointegration of dental implants [75]. 

All the studies addressing the direct and indirect effects of AGEs in periodontal ligament tissue and in peri-implantitis are summarized in Table 1.

## 5. AGE-Targeted Therapeutic Approaches in Periodontitis

The major therapeutic strategy for the attenuation of periodontal disease used to rely on the mechanical removal of dental biofilms from the surfaces of hard oral tissues. New data, nonetheless, offer a completely new perspective as the composition of experimental toothpastes may contain specific salutary substances, such as hyaluronic acid, parabiotic, and lactoferrine, which in parallel with the biofilm removal improve oral health [76]. In future, toothpastes containing AGE blockers could be a helpful means of countering periodontal inflammation provoked by AGE accumulation. Another novel root of administration of the drug is the irrigation of periodontal pockets with a water-based mixture. For instance, ozonated water applied inside the periodontal pockets periodically for some minutes was shown to improve clinical indices of inflammation, especially in acute and necrotizing periodontal diseases [77]. Furthermore, recent in vitro and in vivo studies have detected several experimental agents targeting AGE levels.

### 5.1. In Vitro Studies

Glucagon-like peptide-1 (GLP-1) presents a novel agent able to ameliorate the osteogenic potential, which is negatively influenced by the presence of AGEs. GLP-1 can attenuate RAGE expression and protein kinase Cβ2 phosphorylation, thus interfering with AGEs action in hPDLSCs [78]. Analogous to GLP-1, dickkopf Wnt signaling pathway inhibitor 1 (DKK-1) could rescue the potential of hPDLSCs, promote bone formation in vivo, and decrease RAGE expression [79]. Furthermore, magnolol, a well-studied anti-inflammatory drug, was tested in HGF with a beneficial impact. Magnolol reversed the AGE-induced ROS production in parallel with IL-6 and IL-8 elevation. The underlying molecular mechanism possibly involves the upregulation of nuclear factor-erythroid factor 2-related factor 2 (Nrf2), a factor that is pathologically suppressed by AGEs [18].

### 5.2. In Vivo Studies

Considering the important role of AGEs in periodontal destruction, AGE inhibitors with the ability to reverse the consequences of AGEs accumulation in the periodontal tissues are highly demanded. The initial attempts in this direction included the use of two specific molecules, aminoguanidine (AG) and N-phenacylthiazolium bromide (PTB). The first one is an AGE-specific inhibitor while the second is a glycated cross-link breaker of the extracellular matrix. Both molecules were shown to successfully accelerate wound healing and collagen matrix deposition in 14 days after full-thickness palatal graft harvesting [80]. Similar results were drawn from the use of AG and PTB against experimental periodontitis in rats. AG was shown to interfere with the AGE–RAGE axis by maintaining the tissue integrity and reducing alveolar bone loss during the phase of experimental induction of periodontitis [81]. PTB significantly improved hPDLCs viability by blocking the AGE–RAGE axis. Systemic PTB administration efficiently reduced TNF-α expression and AGE deposition [82]. Moreover, the combination of PTB with a pH-responsive hydrogel for more efficient subgingival delivery of PTB was shown to significantly retard periodontal bone loss in a rat model. The chitosan-based hydrogels may prove a promising alternative method for local delivery of anti-inflammatory agents and for the preservation of periodontal tissue integrity [83]. Recently, more agents have been detected to counteract AGE interactions and accumulation in periodontal tissues. Apart from AG and PTB, vitamin C administration decreased AGE-positive cells, which were abundant in periodontal and diabetic subjects [17]. In a similar way, astraxanthin effectively upregulated the Nrf2 signaling pathway, counteracted the oxidative stress that was attributed to AGEs, and enhanced osteogenesis of hPDLCs in vivo [84]. In this way, the Nrf2 pathway offers an indirect, alternative option to confront the detrimental effects of high glucose levels on the periodontium.

## 6. Conclusions

Taken together, the accumulation of AGEs plays a pivotal role in periodontitis, exerting both direct and indirect effects in periodontal ligament cells through the activation of the AGE–RAGE signaling axis. sRAGE and AGE levels could be used in GCF and serum as novel biomarkers for the assessment and monitoring of the periodontal status. The irreversible accumulation of AGEs in the periodontal tissues is devastating for the viability of periodontal cells such as the fibroblasts and has a negative impact on the bone metabolism of osteocytes. Acting in synergy, DM as well as Pg LPS deteriorate the complications induced by a high concentration of AGEs, indicating that periodontal destruction may be diminished by the parallel regulation of DM and AGE levels and removing the dental plaque. Furthermore, AGEs and the associated oxidative stress are critical risk factors in peri-implantitis, setting the dental implant osseointegration at risk and requiring special attention. Current research efforts are pointing towards the beneficial impact of AGE blockers or inhibitors such as aminoguanidine and N-phenacylthiazolium bromide in hPDLCs through oral or passive administration via toothpastes or water irrigation, indicating a new area of research and drug development for periodontitis. 

## Figures and Tables

**Table 1:** Pre-clinical and clinical studies of AGE effects in periodontal ligament tissues.

Type of Study	Cell Type/Tissue/Biological Fluid	AGE Effects	Reference
In vitro	Rat bone marrow cells	Decreased collagen type I, core-binding protein-a and osteocalcin production Increased IL-1β and S1008A production	[19]
	Mouse osteocytic-like cells	Increased sclerostin, IL-6, and TNF-α production	[20]
	Human gingival fibroblasts	Increase IL-6 and IL-8 production	[21]
	Human osteoblasts	Nicotine enhances AGEs-related actions by increasing H_2_O_2_ production	[23]
	Human gingival fibroblasts	Reduced HGF cell viability and decreased collagen type I and III production	[27]
	Human gingival fibroblasts	Decreased collagen type I production	[28]
	Human gingival fibroblasts and periodontal ligament fibroblasts	Fibronectin and collage type I glycation	[29]
	Human gingival fibroblasts	Increased HGF cell apoptosis	[30]
	Human gingival fibroblasts	Increased MMP-1, MCP-1, IL-6, and IL-8 production	[31]
	Human gingival fibroblasts	Increased IL-6, RAGE, and ICAM-1 production	[32]
	Human periodontal ligament cells	Increased IL-6 and IL-8 production	[36]
	Human periodontal ligament cells	Increased IL-6 and IL-8 production	[37]
	Human periodontal ligament cells	Human periodontal ligament cells autophagy	[38]
	Human gingival epithelial cells	Increased RAGE expression	[39]
	Periodontal ligament stem cells	Increased endogenous ROS	[40]
	Human periodontal ligament stem cells	Less-calcified osteogenic nodules	[41]
	Human periodontal ligament stem cells	Inhibition of osteogenic differentiation	[42]
	Human periodontal ligament cells	Inhibition of differentiation	[43]
	Human monocytes (THP-1 cells)	Increased IL-1β and TNF-α production	[44]
	Gingival fibroblasts	Increased MMP-1 production	[55]
	Periodontal ligament fibroblasts	Increased RAGE and cell apoptosis	[56]
	Human periodontal ligament cells	Increased IL-6, IL-1β, NLRP1, and NLRP3 production	[58]
	Human oral epithelial cells	Increased LCN-2 production	[59]
	Human gingival fibroblasts	RAGE expression increased by nornicotine	[62]
	Vascular endothelial cells	Increased MCP-1 production	[67]
In vivo	Serum	Secretion of MMP-1, increased IL-17 expression, and decreased beta-defensine-3 production	[33]
	Gingival connective tissue	Increased oxidative stress and IL-6 production	[34]
	Human periodontal ligament cells	Increased CSF-1 and VEGF production	[35]
	Gingival tissues	Anti-inflammatory effect of sRAGE	[47]
	Wound healing assessment	Anti-inflammatory effect of aminoguanidine	[48]
	Periodontal tissues	Increased IL-1 and TNF-α production	[50]
	Dendritic cells and osteoclasts	Reduction in dendritic cell migration	[52]
	Human periodontal ligament cells and mesenchymal stem cells	Increased RAGE and TLRs production	[57]
	Cultured human endothelial cells and murine vasculature	Increased VCAM-1 production	[68]
Clinical	GCF	Increased AGEs levels in T2DM patients	[24]
	GCF	Did not significantly increase AGEs levels between T1DM patients and healthy	[25]
	Serum	Increased AGEs levels in T2DM patients	[26]
	Gingival tissues	Increased RAGE levels in T2DM patients	[46]
	Periodontal tissues	RAGEG82S gene polymorphism as a risk factor of periodontitis	[51]
	Saliva, serum	Increased AGEs levels in periodontal patients rather than non-periodontal	[53]
	Serum, GCF	Increased TNF-α and reduced sRAGE levels	[54]
	Gingival tissues	Increased RAGE levels in DM patients	[60]
	Gingival tissues	Main location of RAGE is the basal epithelial membrane	[61]
	Gingival tissues	Anti-inflammatory effect of AGER1	[63]
	Gingival tissues	Increased RAGE levels in gingival tissues of smokers	[64]
	Peri-implant tissues	Increased RAGE levels	[65]
	Peri-apical tissues	Increased RAGE levels	[66]
	Saliva and apical-coronal tissues	Increased AGEs levels and oxidative stress	[71]
	Saliva, peri-implant tissues, and periodontal tissues	Increased AGEs levels and oxidative stress	[72]
	PISF	Increased AGEs levels in T2DM patients	[73]
	PISF	Increased AGEs levels in T2DM patients	[74]
	PISF	Increased AGEs levels in smokers	[75]

## Abbreviations

AG	aminoguanidine
AGER1	AGE receptor 1
AGEs	Advanced Glycation End Products
ALP	alkaline phosphatase
CSF-1	colony stimulating factor-1
DM	Diabetes Mellitus
DM1/DM2	Type 1 Diabetes Mellitus and Type 2 Diabetes Mellitus
ERK	extracellular signal-regulated kinase
GCF	gingival crevicular fluid
HbA1c	hemoglobin A1c
HGF	human gingival fibroblast
hPDL	human periodontal ligament
hPDLSCs	human periodontal ligament stem cells
IL-1β, 6, 10	interleukins 1β, 6, 10
JNK	c-Jun NH2-terminal kinase
LCN2	neutrophil gelatinase-associated lipocalin
MAPK	mitogen-activated protein kinase
MCP-1	monocyte chemoattractant protein-1
MG-H1	hydroimidazolone
MMP	matrix metalloproteinase
NF-κB	nuclear factor kappa-light-chain-enhancer of activated B cells
NLRP1,3	nucleotide-binding domain leucine-rich repeat proteins 1,3
NOD2	nucleotide-binding oligomerization domain-containing protein 2
Pg LPS	*Porphyromonas gingivalis lipopolysaccharide*
PISF	peri-implant sulcus fluid
RAGE	receptor for AGEs
RANKL	receptor activator of nuclear factor κΒ ligand
ROS	reactive oxygen species
S100A8,9	S100 calcium-binding protein A8,9
sRAGE	soluble RAGE
TLR	Toll-like receptor
TNF-α	tumor necrosis factor-α
VEGF	vascular endothelial growth factor
